# Prevalence of depression in adults with type 2 diabetes in the Basque Country: relationship with glycaemic control and health care costs

**DOI:** 10.1186/1471-2458-14-769

**Published:** 2014-07-30

**Authors:** Edurne Alonso-Morán, Altynai Satylganova, Juan F Orueta, Roberto Nuño-Solinis

**Affiliations:** O+berri, Basque Institute for Healthcare Innovation, Torre del BEC (Bilbao Exhibition Centre), Ronda de Azkue 1, 48902 Barakaldo, Spain; Reghealth, European Master in Sustainable Regional Health Systems, Latviu gatve 15, Vilnius, Lithuania; Osakidetza, Basque Health Service, Centro de Salud de Astrabudua, Mezo 35, 48950 Erandio, Spain

**Keywords:** Depression, Type 2 diabetes mellitus, Glycaemic control, Healthcare costs, Comorbidity

## Abstract

**Background:**

The aim of the study was to estimate the prevalence of depression in the population diagnosed with diabetes type 2 and to test the hypothesis that the presence of depression in such cases was associated with a) worse glycaemic control, and b) higher healthcare costs.

**Methods:**

We conducted a cross-sectional analysis, from 1st September 2010 to 31st August 2011, among patients with type 2 diabetes aged 35 years and over in the Basque Country. It was identified how many of them had also depression. The database included administrative individual level information on age, sex, healthcare costs, other comorbidities, and values of glycaemic control (HbA_1c_). Deprivation index variable was used as socioeconomic measure and, to observe the coexistent pathologies, all the patients diagnoses were categorized by Adjusted Clinical Groups. We used a measure of association, a logistic and a linear regression for analysis.

**Results:**

12.392 (9.8%) of type 2 diabetes patients were diagnosed with depression, being the prevalence 5.2% for males and 15.1% for females. This comorbidity was higher among the most deprived population. There was no association between the presence of depression and glycaemic control. We estimated that the comorbidity average cost per patient/year was 516€ higher than in patients with just type 2 diabetes (P < 0.001) adjusted by the other covariates.

**Conclusions:**

We did not find any relationship between depression and glycaemic control in patients with type 2 diabetes. However, the comorbidity was associated with significantly high healthcare costs compared to that of type 2 diabetes occurring alone, after adjusting by other illness. Thus, there is a need of more precise recognition, screening and monitoring of depression among diabetic population. Evidence-based treatment for depression should be included in type 2 diabetes clinical guidelines.

## Background

The social and economic burden of type 2 diabetes is a matter of concern for many health systems worldwide. According to a recent study, the prevalence of type 2 diabetes in Spain is 13.8% in the population above 18 years old [1]. Moreover, incidence and prevalence of diabetes mellitus along with other chronic diseases is constantly growing [2] and in recent decades, this problem is exacerbated by the presence of other chronic conditions [3], including chronic mental disorders such as depression [4].

Depression is the most common mental disorder and generates a great impact on the individual, family and society in terms of suffering, disability and economic costs, a phenomenon that seems to occur in many parts of the world. Depression is estimated to affect 350 million people worldwide [5] and it also causes a large increase in all-cause mortality risk (up to 70%) [6], at the same time being the most expensive mental disorder in Europe [7].

According to several studies, presence of additional diagnosis of depression in patients with type 2 diabetes can negatively influence control, treatment and outcomes of both diseases [8]. According to World Health Surveys [9] conducted in 2007, combination of two or more chronic conditions with depression obtained the lowest scores making diabetes and its complications the worst combination for depression (comparing it to combination of depression with other chronic conditions such as asthma or angina), in terms of the worst quality of life and disability.

However, data about relations of diabetes and depression ranges widely, depending on the type of diabetes and the fact whether depression is self-reported or clinically justified [10]. Moreover, the influence of one disease in the aetiology of the other is most likely to have bidirectional character [11, 12]. Depression as a baseline disease can increase incidence of type 2 diabetes through increasing vulnerability to various type 2 diabetes associated factors. Latest meta-analysis [13] of comorbidity of these two diseases suggests that depression and diabetes may co-occur due to shared biochemical changes in hormonal system and psychological burden of chronic disease. Another study reported that the prevalence of depression among patients with type 2 diabetes is twice than in general population, 19.1% and 10.7% respectively [14].

Several studies have shown that the following socio-demographic associated factors can be predictive of having depression among type 2 diabetes patients: female sex, younger age, lower socioeconomic status, being unmarried, poor social support, certain ethnicities [9]. Furthermore, depression is associated with non-adherence to diabetes self-care such as following dietary restrictions, medication compliance, and blood glucose monitoring, resulting in worse overall clinical outcomes [11].

In time of recent years, there have been many studies presenting evidence that comorbid depression among individuals with type 2 diabetes is associated with poor diabetes outcomes. A meta-analysis of 24 studies found that depression was significantly associated with poor glycaemic control in individuals with type 2 diabetes [15]. Richardson *et al.* [16] found that in over 4 years of follow-up there was a significant longitudinal relationship between depression and glycaemic control, and that depression was associated with persistently higher HbA_1c_ levels over the time period. Other review detected that diabetes self-care did not fully account for the relationship between depression and HbA_1c_ levels [17].

In economic terms, recent studies indicate increased healthcare utilization and healthcare costs among individuals with comorbidity of diabetes and depression. One study found that patient with diabetes and depression had higher diabetes-related total medical costs (14,253€) than patients with diabetes alone (3,559€) [18]. Another study showed that individuals with diabetes and depression had 2 times higher health care costs compared with those who did not have depression [19].

This study aims to estimate the prevalence of depression in the population diagnosed with type 2 diabetes. Furthermore, to establish the relationship between depression as comorbidity in patients with type 2 diabetes and glycaemic control, and to test the impact on healthcare cost.

## Methods

The study protocol was approved by the Ethical Committee of Clinical Research of Euskadi (PI2014074), Spain. The informed consent was not obtained because patient health record was anonymized and de-identified prior to analysis.

### Data source and design

We conducted a descriptive cross-sectional analysis in which we used the database of the population stratification program of the Basque Health Service – Osakidetza [20]. Although this programme began in 2010, with the aim of classifying all inhabitants of the autonomous region in terms of their future healthcare needs, the database (PREST) contains information collected since 2007 and includes every citizen covered by the public health insurance in the Basque Country, regardless of whether they had made any contact with or use of the Basque Health Service. That is, practically all the inhabitants of the Basque Country are included.

PREST was assembled combining information on individuals from several sources (primary and specialized health care records, computerised files from day hospitals, emergency departments’ registries, hospital discharge reports, and census data) and involves the following data:

- Demographic data: age, sex and census area of residence.
- A register for any contact of the patients with Osakidetza, containing the type of service provided, codes for diagnoses and information on significant procedures (such as dialysis, radiation therapy, chemotherapy or rehabilitation therapies).
- Prescriptions in primary care.

Services for which no information was available comprise: non-acute Mental Health (both admissions and outpatient visits), hospital-at-home services, medical transport, prostheses and other equipment delivered to patients at home.

In Spain, according to the Health Ministry’s regulation [21], diagnoses and procedures are codified by means of the ICD-9-CM system [22], while the Anatomical Therapeutic Chemical coding system (ATC) [23] is used for drugs prescription.

Taking into account all this information, Basque citizens are annually classified using the Johns Hopkins Adjusted Clinical Groups (ACGs) case-mix system [24]. Such system enables health problems to be identified from diagnoses and prescriptions and patients to be categorized according to their health care needs.

Using the above mentioned resources, the observed population consisted of patients who have been covered by the Basque Country’s public health insurance for at least 6 months prior to period of study (dated from 1st of September in 2010 to 31st of August in 2011), independently if patients had visited doctors or health centers of Osakidetza during such period.

So, firstly, we identified diabetes mellitus patients who were ≥ 35 years old because diabetes type 2 is uncommon at younger ages [25]. It was considered to have diabetes mellitus, those who had either being prescribed with oral anti-diabetics drugs or had any kind of diabetes-related diagnose or its complications, before 31st of August 2011; of them were excluded all people who had one or more diagnosis related to type 1 diabetes or with non-specified diabetes treated exclusively with insulin.

Secondly, we identified those patients with depression diagnosed either in Primary Care and hospital settings. Often these health professionals do not perform in their diagnoses a description precise enough to allow differentiation between different types of depressive disorders and, therefore, we accepted the presence of any of them. Since the database contains information of a period of 4 years, in order to exclude those episodes that are not currently active, we looked only for patients who had a diagnosis of depression in last 12 months or had repeated prescriptions of antidepressants during at least four months in the last year.

### Variables and analysis

Since many people had more than one measurement of glycated haemoglobin, the last measurement registered in the period analyzed was used. The values of HbA_1c_ were divided into four groups according to the study of Berkowitz *et al.* [26], these groups were: ≤7% (group 1), between >7% and ≤8% (group 2), between >8% and ≤9% (group 3) and finally >9% (group 4).

In order to take into account the presence of comorbidities, we used Aggregated Diagnosis Groups (ADGs), which is the component of the Johns Hopkins ACG case-mix system. ADG consists of 32 categories, specifically designed to aggregate diagnoses into groups with similar severity, duration of condition, and treatment needs. A more complete description of this methodology can be found in the bibliography [24].

The deprivation index of the census tract (median population size is equal to 1,200 inhabitants) of residence proposed by the MEDEA project [27] was used as a proxy for individual socioeconomic position. Its calculation takes into account the percentages of residents who are manual workers, unemployed, temporary employees, or have not finished primary school, overall and also specifically among young people, given the most recent Census available. According to such index, tracts are ordered and categorized into quintiles (the fifth one corresponds to the most deprived areas and the first one to the less unfavourable conditions). This index is not specific to the ages groups observed in this study; however, it provides a measure of the level of access to material and social resources in a community and has been shown to be correlated with general rates of mortality [28] and morbidity, even in particular population groups, as the elderly [29]. Although been elaborated in 2008 (from Census data collected in 2001), and from then it is likely that the values of the observed variables percentages have experienced variations, it is conceivable that the relative position of each area in respect to the others have not suffered relevant alterations; in fact, in recent years it has been proved that the deprivation index is related to the health of the resident inhabitants [30, 31].

Health care utilization costs were also estimated for the period of study. For variables such as visits to Accident & Emergency (A & E), rehabilitation sessions, outpatient care, primary care visits, laboratory tests and radiological examinations ordered by primary care, and various outpatient procedures (dialysis, radiotherapy and chemotherapy); the number of services used by each patient was multiplied by standardized costs (the average cost of each service provided to a patient treated in Osakidetza, according to calculations made by the aforementioned organisation). We estimated healthcare costs of primary care prescriptions recorded in electronic health records based on the market value of the drugs. The costs of hospitalisation and outpatient surgery were calculated in relation to their cost-weights in the corresponding Diagnosis-Related Groups (DRGs).

Means and standard deviation of continuous variables and frequencies of ordinal or categorical variables were calculated during the study, stratifying by sex and age band. Statistical significance test (test of Pearson *X*^2^) was calculated to establish the relationship between presence or absence of comorbidity and other variables such as sex, age band, socioeconomic level and glycaemic control. Logistic regression analysis was performed to see the associated factors to depression in patients with diabetes type 2 using as independent variables HbA_1c_ categories, sex, age band, ADGs and deprivation index. Finally, linear regression was used to see how many, on average, of the total cost is imputable to depression, HbA_1c_ categories, sex, age band, ADGs and deprivation index.

Analysis was conducted on STATA, Data Analysis and Statistical Software, Release 12 (StataCorp, LP, College Station, TX).

## Results

Among all the population of Basque Country who were 35 or more years old (N = 1,473,937), 126,894 were diagnosed with type 2 diabetes (prevalence 8.6%, CI 95% [8.6, 8.7]) and 76,594 with depression (prevalence 5.2%, CI 95% [5.16, 5.23]). The prevalence of depression among patients with type 2 diabetes was 9.8% (CI 95% [9.6, 9.9]). Comparing both sexes, the prevalence was 5.2% (CI 95% [5.0, 5.3]) for men and was 15.1% (CI 95% [14.9, 15.4]) for women. There was a relationship between gender and the comorbidity (p value of Pearson *X*^2^ < 0.001). The mean age between patients who have the comorbidity was 72.9 (CI 05% [72.7, 73.1]).

Among patients with type 2 diabetes and depression, we could follow up a gradual growth of depression prevalence from the most affluent layers of population to the most deprived (see Table 1). The difference between the richest quintile and the poorest quintile was around a 1% and there was a relationship between socioeconomic level and the comorbidity (p value of Pearson *X*^2^ < 0.001) for the total, males and females (p < 0.001). We could also see an increasing gradient in patient’s distribution by age group, being more prevalent in patients aged ≥ 65 years than in patients aged < 65 years for diabetes type 2 and for the comorbidity. Also, there was a relationship between age bands and the comorbidity (p value of Pearson *X*^2^ < 0.001).

**Table 1:** **Distribution of patients with diabetes type 2 and with comorbidity (depression and type 2 diabetes)**

		Males		Females	
		N	%	N	%
Patients with type 2 diabetes mellitus	Age band				
	Age < 65	25,813	39.8	12,771	25.8
	Age ≥ 65	39,122	60.3	36,796	74.2
	Deprivation index				
	1	10,757	16.6	7,267	14.7
	2	13,032	20.1	9,207	18.6
	3	13,402	20.6	9,980	20.1
	4	13,953	21.5	10,970	22.1
	5	13,791	21.2	12,143	24.5
	Total	64,935		49,567	
	HbA_1c_				
	1 (≤7%)	32,193	65.4	23,620	62.4
	2 (>7% & ≤8%)	10,148	20.6	8,535	22.5
	3 (>8% & ≤9%)	3,847	7.8	3,236	8.6
	4 (>9%)	3,005	6.1	2,479	6.6
	Total	49,193		37,870	
Patients with type 2 diabetes mellitus and depression	Age band				
	Age < 65	1,058	29.8	1,736	19.6
	Age ≥ 65	2,488	70.2	7,110	80.4
	Deprivation index				
	1	618	17.4	1,239	14.0
	2	701	19.8	1,650	18.7
	3	736	20.8	1,799	20.3
	4	747	21.1	1,883	21.3
	5	744	21.0	2,275	25.7
	Total	3,546		8,846	
	HbA_1c_				
	1 (≤7%)	1,805	65.3	4,491	63.2
	2 (>7% & ≤8%)	544	19.7	1,554	21.9
	3 (>8% & ≤9%)	236	8.5	574	8.1
	4 (>9%)	179	6.5	487	6.9
	Total	2,764		7,106	

The Table 1 shows the distribution of these patients by range of values of HbA_1c_. The test was carried out in the 76.04% of the patients with diabetes type 2 and without depression and in the 79.65% of the patients with the comorbidity, being the differences between both proportions statistically significant (p < 0.001). Glycaemic control was better in men than women in all defined groups and in both populations. The control of HbA_1c_ between patients with diabetes type 2 and patients with the comorbidity was very similar.

Results of analysis of statistical significance between presence or absence of depression and HbA_1c_ values among patients with type 2 diabetes showed that there was not a general association (Pearson chi2: p = 0.365).

Logistic regression analysis was performed to see if depression in type 2 diabetes population was associated with the following factors: HbA_1c_, sex, age band, ADG and deprivation index. For every man, 2.7 women suffered comorbidity (OR = 2.7; CI 95%: 2.6-2.9). For every person under 65 years, 1.5 people aged ≥ 65 years suffered it (OR = 1.5; CI 95%: 1.4-1.6). However, glycaemic control and socioeconomic level were not associated factors of having depression (see Table 2).

**Table 2:** **Logistic regression with odds ratios and confidence intervals at 95% represented**

	Depression	
Characteristics	Odds ratio	95% confidence interval
HbA_1c_		
HbA_1c_ 1 (referent)	1	
HbA_1c_ 2	0.99	0.93, 1.05
HbA_1c_ 3	1.01	0.93, 1.02
HbA_1c_ 4	1.11	0.99, 1.19
Sex		
Males (referent)	1	
Females	2.71	2.58, 2.86
Deprivation index		
Deprivation index 1 (referent)	1	
Deprivation index 2	0.95	0.88, 1.03
Deprivation index 3	1.01	0.93, 1.09
Deprivation index 4	0.93	0.86, 1.01
Deprivation index 5	0.96	0.89, 1.03
Age band		
Age band < 65 (referent)	1	
Age band ≥ 65	1.52	1.44, 1.61

This regression analysis was conducted with having depression as comorbidity as dependent variable and HbA_1c_, sex, deprivation index, age band and ADGs as independent variables. As the ADGs are 32 variables, they are not represented in this table, if you want to know the exact values, please contact to the authors.

A regression analysis was performed in order to see if there were statistically significant differences in mean cost between groups with and without comorbidity of depression (see Table 3). The *R*^2^ was 0.41. *R*^2^ is a statistic that gives information about the goodness of fit of a model. In regression, the *R*^2^ coefficient of determination is a statistical measure of how well the regression line approximates the real data points. An *R*^2^ of 1 indicates that the regression line perfectly fits the data. Then, independent variables explained the 41.2% of the total variance.

**Table 3:** **Linear regression analysis with coefficients and confidence intervals at 95%**

Total cost		
Characteristics	Coefficients	95% confidence interval
HbA_1c_		
HbA_1c_ 1 (referent)		
HbA_1c_ 2	259.1	195.6, 322.6
HbA_1c_ 3	565.6	470.8, 660.4
HbA_1c_ 4	441.7	335.4, 548.0
Sex		
Males (referent)		
Females	-179.5	-233.6, -125.3
Depression		
No (referent)		
Yes	515.9	424.2, 607.6
Deprivation index		
Deprivation index 1 (referent)		
Deprivation index 2	30.0	-58.7, 118.7
Deprivation index 3	90.7	3.4, 178.1
Deprivation index 4	116.8	30.6, 203.0
Deprivation index 5	73.2	-12.5, 158.8
Age band		
Age band < 65 (referent)		
Age band ≥ 65	160.1	103.0, 217.2

This regression analysis was made with total cost as dependent variable and HbA_1c_, sex, age band, having depression as comorbidity, deprivation index and ADGs as independent variables. As the ADGs are 32 variables, they are not represented in this table, if you want to know the exact values, please contact to the authors.

The age band and sex were statistically significant in the model. People who were ≥ 65 years spent 160€ more than people who were under 65 years and women spent 179.5€ less than men. In addition, a bad control of HbA_1c_ (HbA_1c_ >7%) and the depression as comorbidity in type 2 diabetes patients increased the total mean cost, being statistically significant for each group. Finally, the group 2 and 3 of the deprivation index spent more than the group 1, 90.7€ and 116.8€ more than group 1 respectively.

## Discussion

In this study, the prevalence of depression among patients with type 2 diabetes was 9.8%, 5.2% for men and 15.1% for women. This comorbidity was more prevalent in the most deprived areas and this prevalence increased with the age. Our results reported similar prevalence rate of depression among patients with type 2 diabetes compared to other published studies. A meta-analysis of 42 published studies found that the prevalence of major depression among patients with diabetes type 2 was 11% [10] and Roy T. *et al.* [14] reported a prevalence of depression two times higher among patients with type 2 diabetes than in general population. In relation to gender, this meta-analysis [10] also showed high prevalence of depression among females. These differences in prevalence among the studies are due to the different methods applied. In addition, Nichols *et al.* [32] found that people with diabetes and comorbid depression were more likely to be women, had lower incomes and health status, and more diabetes complications. Other study showed that age, ethnicity and monthly household income were significantly associated with depression [33]. So, in line with other researchers, we also found that female sex and age was related to depression as comorbidity.

Our logistic analysis has shown that for every man who suffered comorbidity, there were 2.7 women who suffered it, which was consistent with the literature. For instance, a meta-analysis [10] showed that the combined prevalence was significantly higher in women with diabetes than in men with diabetes (OR = 1.6, 95% CI: [1.4, 1.8]).

Our study showed that poor glycaemic control was not associated with a higher prevalence of depression. It is consistent with other studies that did not find significant relationship between glycaemic control and potential for depression [34–36]. However, Lustman *et al.* have found that depression was significantly associated with poor glycaemic control in individuals with type 1 and type 2 diabetes [15] and Richardson *et al.* assessed the longitudinal effects of depression on glycaemic control [16]. Moreover, cross- sectional studies found a significant relationship in patients with type 1 diabetes but no in patients with type 2 diabetes [37, 38].

The total cost was associated with a bad control of HbA_1c_, sex, age and depression as comorbidity in patients who have diabetes type 2. Some studies of healthcare utilization and healthcare costs confirm that the coexistence of depression among individuals with diabetes is associated with greater healthcare service utilization and costs [18, 19, 32, 39].

In our study the comorbid women accounted for less healthcare costs than men. In study carried out by Burns *et al.,* total health care costs were higher for males than females [40]. In addition, Orueta *et al.* [31] demonstrated that once adjusted per number of chronic conditions and socioeconomic characteristics, individual cost for females was lower than for males for all ages but in the range of 18–44 years old, as expected due to obstetric care.

Our study showed that, on average, people with depression have spent 516€ more than people without depression. Since, regression included ADGs which control the burden of diseases; this difference could be really attributed to depression (other illness which can have patients are control by these variables). So, this difference could be explained by number of reasons such as cost implications of depression treatment, higher occurrence and consequent cost of type 2 diabetes complications in comorbid group [41].

Most authors recognize that depression and diabetes might have linked pathogenic pathways. Oladeji *et al.* [42] discussed in their recent review, that depression could be explained through the following associated factors, among others: macrovascular and microvascular complications of diabetes, disability and presence of comorbidities, perceived burden of diabetes, length and treatment of diabetes, smoking and persistent poor glycaemic control.

A current meta-analysis on bidirectional relationship of diabetes type 2 and depression [13] found that relative risk increase in incidence of depression in diabetic patients and vice versa can be associated with biochemical changes that occur during primary disease. They suggest that due to small relative and absolute risk differences in groups with or without one of comorbidities, they might share common causes and/or risk factors rather than be associated with each other.

One of the strengths of our study was defined by the fact that Osakidetza is a tax-funded National Health System that offers universal coverage to all citizens. This gave us the possibility to include the entire known diabetic population in the geographical area of our study, thus avoiding selection bias. Moreover, it included cases of depression that were clinically diagnosed or estimated through prescriptions because a study carried out in the Basque Country [43] showed that the prevalence calculated using this combined sources of data was higher than the obtained from self-reported data in Basque Health Survey.

Also, the data was consistent with previous findings and utilized a database containing information about primary care, specialized, outpatient hospital care as well as prescriptions. This is relevant as other authors have established that the use of a single source can produce inaccurate calculations [44, 45], while the complementary use of various sources contributes to a better description of the health problems of people [43]. Finally, all regressions were controlled through inclusion ADGs in our analysis, variable that controls the burden of diseases.

The limitations of this study were that administrative databases only contained information about conditions for which people looked for medical attention. Therefore, the prevalence of diabetes and depression may have reflected only known cases, and excluded the presence of the cases, that were not known by patients or doctors. Furthermore, antidepressants can be used for other different pathologies, not only depression, which could lead to the misidentification of some patients. Moreover, in this study, the age was limited to equal or more than 35 years and thus we have not estimated the prevalence of depression among patients with diabetes type 2 in younger ages. Ganasegeran *et al.* [33] have showed that this prevalence is higher in older ages but Zhao *et al.* [46] have demonstrated that diabetes is significantly associated with depression, particularly in young adults.

Also, the cost was calculated in relation to the total cost of care provided to patients with diabetes type 2, and it incorporated all the costs related to care of the disease, but also to other conditions not necessarily related to it. The non-acute mental health services are not included. These services account for 2% of the budget of the Basque Health Service and that have not been considered in our cost estimations.

Another limitation refers to the social variable used (deprivation index) which, given its ecological character, may underestimate the contribution of individual socioeconomic characteristics. Such index was used to classify geographic areas in quintiles and, although is probable that actual data relative to unemployment and others have changed since last census, we consider that the relative situation of each area with respect to the other ones has not suffered a substantial alteration. Finally, we had not access to the type of antidepressants used for treatment in our study population. Basset *et al.* [47] also discuss hyperglycaemic effects of some antidepressants on diabetes type 2 and thus further research on types of antidepressants used and their effects on glycaemic control could be an interesting consideration for future.

In last decade, there has been significant growth in research of association of physical and mental disorders, as well as holistic approach to their treatment [10, 48, 49]. However, this approach still has not been very well translated to the everyday clinical practice, considering that diseases are being treated separately in a single-disease framework, which influences patient adherence to treatment and clinical outcomes.

## Conclusions

Our research has allowed us to obtain an accurate picture of the prevalence of depression among patients with diabetes type 2 in the Basque Country. It has also allowed us to test the relationship between glycaemic control and prevalence of depression as comorbidity, and its influence to healthcare costs. Based on our research we can conclude that poor glycaemic control is not directly associated with prevalence of depression as comorbidity, but it can be an important factor of healthcare costs.

Findings of our research, along with results of our literature overview, highlight the importance of more precise recognition of depression among diabetic population and provision of periodic screening and monitoring for depression among type 2 diabetes patients. Moreover, evidence-based treatment of depression for diabetic patients should be included in type 2 diabetes clinical treatment guidelines. There is evidence supporting the cost-effectiveness [50] of interventions tackling type 2 diabetes and depression through “collaborative care” models.

## Abbreviations

PREST: Basque Country Stratification Program
ACGs: Adjusted Clinical Groups
ADGs: Aggregated Diagnosis Groups
DRGs: Diagnosis Related Groups.
